# Ferroptosis: a double-edged sword mediating immune tolerance of cancer

**DOI:** 10.1038/s41419-022-05384-6

**Published:** 2022-11-05

**Authors:** Qin Dang, Ziqi Sun, Yang Wang, Libo Wang, Zaoqu Liu, Xinwei Han

**Affiliations:** 1 Department of Interventional Radiology, The First Affiliated Hospital of Zhengzhou University, Zhengzhou, Henan 450052 China; 2 Department of Colorectal Surgery, The First Affiliated Hospital of Zhengzhou University, Zhengzhou, Henan 450052 China; 3 Department of Hepatobiliary and Pancreatic Surgery, The First Affiliated Hospital of Zhengzhou University, Zhengzhou, Henan 450052 China

**Keywords:** Immunosurveillance, Cell death

## Abstract

The term ferroptosis was put forward in 2012 and has been researched exponentially over the past few years. Ferroptosis is an unconventional pattern of iron-dependent programmed cell death, which belongs to a type of necrosis and is distinguished from apoptosis and autophagy. Actuated by iron-dependent phospholipid peroxidation, ferroptosis is modulated by various cellular metabolic and signaling pathways, including amino acid, lipid, iron, and mitochondrial metabolism. Notably, ferroptosis is associated with numerous diseases and plays a double-edged sword role. Particularly, metastasis-prone or highly-mutated tumor cells are sensitive to ferroptosis. Hence, inducing or prohibiting ferroptosis in tumor cells has vastly promising potential in treating drug-resistant cancers. Immunotolerant cancer cells are not sensitive to the traditional cell death pathway such as apoptosis and necroptosis, while ferroptosis plays a crucial role in mediating tumor and immune cells to antagonize immune tolerance, which has broad prospects in the clinical setting. Herein, we summarized the mechanisms and delineated the regulatory network of ferroptosis, emphasized its dual role in mediating immune tolerance, proposed its significant clinical benefits in the tumor immune microenvironment, and ultimately presented some provocative doubts. This review aims to provide practical guidelines and research directions for the clinical practice of ferroptosis in treating immune-resistant tumors.

## Facts

1. Ferroptosis is a programmed cell death induced by iron-dependent lipid peroxidation, which is modulated by various cellular metabolic and signaling pathways.
2. Ferroptosis has been linked to various diseases and acts as a double-edged sword while mediating immune tolerance.
3. Drug-resistant cancer cells, especially those in a mesenchymal state and prone to metastasis, are remarkably sensitive to ferroptosis, which could bring a promising strategy for drug-resistant cancer treatment.

## Open questions

1. The mechanisms and vulnerability of ferroptosis in varied tumor cells demand further investigations to target precisely and maximize clinical therapeutic benefits.
2. How to modulate immune tolerance by inducing or suppressing cellular ferroptosis in an artificially regulated manner still should be explored.
3. Discovering a method to balance the dual role of ferroptosis in tumor cells, anti-tumor immune cells, and immunosuppressive cells is especially crucial to broaden the application of ferroptosis in anti-cancer treatment.
4. Currently whether ferroptosis is immunogenic and whether mitochondria play an essential role in all forms of ferroptosis or only GPX4 inhibition-induced ferroptosis are controversial and still require experimental demonstrations in the future.

## Introduction

Cells serve as the fundamental organizational unit of life. Their multiple activities, including proliferation, differentiation, and ultimately cell death, are essential for ontogeny, homeostasis, and disease progression [1]. Among them, cell death is particularly crucial. Historically, biochemists believed that cell death was unregulated. However, numerous experimental evidence that cell death could be regulated has been accumulating over the past few years [2], and regulated cell death (RCD) was found to rely on specific genetically encoded molecular mechanisms driving the targeted elimination of excess and irreversibly injured or/and underlying detrimental cells [3]. Ferroptosis, a term firstly recovered in 2012, is a form of RCD that relies on iron, driven by unrestricted lipid peroxides [4]. Given the reliance on oxygen as the final electron acceptor in reduction/oxidation (redox)-based metabolic processes, the critical association with the cell destiny decisions lies in the way cells address oxidative stress [5–7]. Although ferroptosis was disclosed in mammalian research, this inimitable way of RCD could also be observed in distant species [8–10]. Hence, ferroptosis is potentially one of the most prevalent and archaic RCD. Marked by the oxidative modification of phospholipid membranes, ferroptosis is distinct from other types of RCD in morphology and mechanism [11, 12]. Cellular morphological characteristics of ferroptosis include: (1) small cellular volume with normal nucleus volume; (2) the absence of pyknosis; (3) atrophic mitochondria with elevated membrane density and diminished mitochondrial crista, instead of typical apoptotic features [11]. In addition, cardinally characterized by a fatal accumulation of lipid peroxides, ferroptosis involves a confrontation between its inducers and defense systems. Once the anti-ferroptosis systems are compromised, ferroptosis occur consequently [13–19]. Thus, ferroptosis is mechanistically distinguished from other forms of RCD that depend on cell death executioner proteins, including gasdermin D-mediated pyroptosis and caspase-mediated apoptosis [20]. As a new concept, the mechanisms behind ferroptosis have only been approximately unveiled in recent studies. The consumption of cysteine leads to the limited synthesis of intracellular reduced glutathione (GSH) [11]. On the basis of optimum activation of glutathione peroxidase 4(GPX4), GSH is required to protect from ferroptosis [21]. GPX4 is a selenoprotein that contributes to efficient phospholipid peroxidation [13, 19, 22]. Consequently, with the depletion of the intracellular pool of GSH, ferroptosis could be triggered under the unrestrained lipid peroxidation [23].

During the neoplastic process, tumor cells obtain immune tolerance to elude immunity, thereby contributing to the defeat of targeted therapy [24]. Immune tolerance is a phenomenon in which immune cells under the antigenic stimulation can’t be activated and generate specific immune effector cells or antibodies, ultimately failing to establish a well-balanced immune response [25]. CD8 + T cells experience transcriptional and epigenetic changes under the impact of the tumor microenvironment (TME) and chronic antigenic stimulation, allowing their failure to generate effector molecules and to acquire a gene expression program associated with depletion, in which the transcription factors Tox and Eomes are identified as force depletion regulatory transcription factors [26, 27]. During this procedure, molecules engaged in modulating T cell tolerance, such as PD-1 and Nr4a1, are particularly crucial [28]. In previous studies, the mechanisms of immune tolerance were widely divided into innate and adaptive tolerance, while the latter can be comprehensively categorized into central and peripheral tolerance [29]. Co-stimulatory or co-inhibitory signals pathway, immune checkpoints, and the presence of specialized cell populations form the basis of peripheral tolerance, which are determinants for modulating the immune response [30–32]. Exorbitant or inadequate co-stimulation can bring about immunotolerance. Immune cells are scrutinized by co-stimulatory or co-inhibitory receptors, specific immune checkpoint inhibitors (ICIs) and Transforming growth factor-β (TGF-β), aiming to impede the devastation of tissues due to immoderate or improper immune responses. Co-inhibitory receptors, including programmed cell death 1 (PD-1) and cytotoxic T lymphocyte antigen 4 (CTLA-4), function as pivotal checkpoints in constraining immune reactions targeting the tumor [33, 34]. Specific immune checkpoint inhibitors targeting PD-1 and CTLA-4 pathways crucially ameliorated the prognosis of patients with multiple types of cancer. TGF-β is a critical enforcer of immunotolerance, curbing the evolvement and activity of the immune system [35]. It can control adaptive immunity via direct promotion of regulatory T cells (Tregs) and suppression of effector T cells and antigen-presenting dendritic cells (DCs). Similarly, TGF-β can also regulate the innate immune system via suppressing natural killer (NK) cells [36]. As regulates both the innate and adaptive immune system, TGF-β is pivotal in tumor immune evasion [37]. Notably, immune tolerance has been a Gordian knot in immunology for several decades. How to reasonably utilize immune tolerance or neutralize the immune tolerance of tumor cells and eventually achieve the therapeutic aims has not been effectively solved.

Ferroptosis could be observed throughout the process of peripheral immunotolerance [38–42]. As a distinct mechanism of RCD, ferroptosis has triggered considerable concern, as addressing ferroptosis might represent new curative options for treating immunotolerant cancers. In this review, we systematically summarized the present cognition of ferroptosis, including its mechanisms and regulatory networks, and deeply analyzed the potential mechanisms of abnormal susceptibility to ferroptosis in certain cancer cells. We also discussed how ferroptosis participates in immune tolerance as a double-edged sword. We aim to provide a refreshing idea for the clinical cancer treatment strategies targeting the dual role of ferroptosis in immune tolerance.

### Ferroptosis

Dolma et al. began high-throughput screening in the early 20th century in search of new small-molecule anti-cancer therapies, and in 2003 published a series of compounds that can induce a different mode of cell death from necrosis and apoptosis [43] (Table 1). In subsequent reverse screening studies, this pattern of cell death was found to be inhibited by iron chelating agents and lipid antioxidants. Therefore, the requirement of iron in such RCD was termed “ferroptosis” by Dixon and coworkers [11].

**Table 1:** Distinctions between ferroptosis and apoptosis.

Forms of RCD	Definition	Characters	Classification	Key molecules	Potential strategies	Targets	Specific targets	Links with immune	References
Apoptosis	Programmed cell death, which is an aumomatically cell death pathway determined by genes.	membrane blebbing, cell shrinkage, condensation of chromatin, and fragmentation of DNA	intrinsic apoptotis	Pro-apoptotic BH3-only proteins(BIM, PUMA, BAD or NOXA), pro-survival BCL-2 proteins(BCL-2, BCL-XL, MCL-1 an BCL-2-related protein A1), BAK, BAX, cytochrome c, SMAC, caspase 3, 7 and 9, XIAP, apoptotic peptidase activating factor 1(APAF1).	Strategies targeting intrinsic pathway	BH3 mimetics	Dual BCL-2 and BCL-XL inhibitors	immunologically silent	[228–237]
							Selective BCL-2 inhibitors		
							BCL-XL inhibitors		
							MCL-1 inhibitors		
						IAP inhibitors and SMAC mimetics	–		
			extrinsic apoptosis	FAS, FAS ligand(FASL), FADD(FAS associated via death domain), death- inducing signalling complex (DISC), caspase 3, 7 and 8, BH3-only protein BID, tBID.	Strategies targeting extrinsic pathway	Death receptor agonists	–		
Ferroptosis	Ferroptosis is trigged by failure in or blockade of the glutathione-dependent antioxidant defences of a cell, which leads to the unrestrained lipid peroxidation and ultimately the killing of the cell.	presence of smaller than normal mitochondria with condensed mitochondrial membrane densities, reduction or vanishing of mitochondria crista, and outermitochondrial membrane rupture, lipid peroxidation, inactivation of GPX4, iron-dependent	Amino acid metabolism	GPX4, GSH, glutamate, cysteine, glycine, GCL, GSS	Inhibiting ferroptosis	The GPX4-GSH system		Dual role in immune tolerance	[44–47, 54–58, 60, 61, 63, 65, 66, 178, 238–241]
						The FSP1-CoQH2 system			
			Lipid peroxidation	ASCL4, LPCAT3		The DHODH-CoQH2 system			
						The GCH1-BH4 system			
			Iron metabolism	TF, TFR, NCOA4, GOT1		The FSP1-Vitamin K system			
					Inducing ferroptosis	GSH depleting compounds			
			Mitochondrial metabolism	AMPK. ACC		GPX4 inhibitors			
						Depletion of GPX4 protein and CoQ10			
						Iron oxidation and indirect GPX4 inactivation			

### Mechanisms governing ferroptosis

#### Amino acid metabolism

**Fig. 1:**
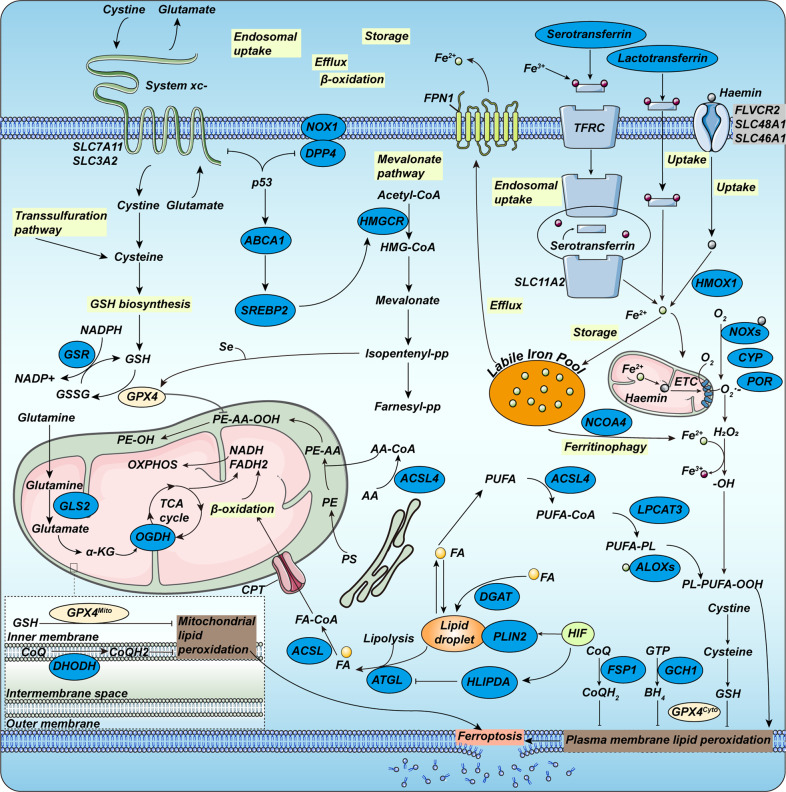
Molecular mechanisms of ferroptosis. Ferroptosis is typically triggered by iron-dependent lipid peroxidation. The cystine/glutamate transporter (also known as system xc-) imports cystine into cells with a 1:1 counter-transport of glutamate. Once inside the cells, cystine can be oxidated into cysteine, which is used to synthesize GSH. Taken as a reducing cofactor, GSH is in the reaction of reducing lipid hydroperoxides to lipid alcohols under the capability of glutathione peroxidase GPX4. Transsulfuration pathway is involved in supporting the availability of cystine and reduced GSH. Respectively, the mevalonate pathway generates a series of biomolecules and then drives ferroptosis. Several proteins (including serotransferrin, lactotransferrin, Transferrin receptor (TFRC), ferroportin 1 (FPN1), nuclear receptor co-activator 4 (NCOA4)) control ferroptosis through the regulation of iron metabolism. Fe^3+^ could be internalized into cells through three distinct pathways including lactotransferrin, haemin and serotransferrin-TFRC-SLC11A2 pathway, during which Fe^3+^ is reduced and storage in the liable iron pool. Cells have evolved at least four systems inhibiting ferroptosis with different subcellular localizations to decrease lipid peroxides. The GPX4-GSH system can collaborate with FSP1-CoQH2 system on the plasma membrane and can also cooperate with DHODH-CoQH2 system on mitochondrial membrane. Of late, the impact of the hypoxia-inducible factor (HIF) system on fatty acid (FA) metabolism has been depicted. α-KG α-ketoglutarate, AA arachidonic acid, ABCA1 ATP- binding cassette subfamily A member 1, ACSL4 Long- chain fatty acid–CoA ligase 4, ATGL adipose triglyceride lipase (also known as PNPL A2), ALOXs Arachidonate lipoxygenases, CoQ coenzyme Q10, CPT carnitine palmitoyl transferase, DGAT diacylglycerol O- acyltransferase, DPP4 dipeptidyl peptidase 4, ETC electron transport chain, ER endoplasmic reticulum, FLVCR2, FPN1 ferroportin 1 (also known as SLC40A1), GLS glutaminase, GSR glutathione disulfide reductase, GSSG glutathione disulfide, HILPDA hypoxia-inducible lipid droplet- associated, HMGCR HMG-CoA reductase, LOX lipoxygenase, LPCAT lyso-phosphatidylcholine acyltransferase, NCOA4 nuclear receptor co-activator 4, NOX1 NADPH oxidase 1, OGDH oxoglutarate dehydrogenase, OXPHOS oxidative phosphorylation, PE phosphatidylethanolamine, PLIN2 perilipin 2, PS phosphatidylserine, SREBP2 sterol regulatory element binding protein 2, system xc- cystine–glutamate antiporter, TFRC transferrin receptor, GCH1 GTP cyclohydrolase 1, HMOX1 Heme oxygenase, SLC48A1 solute carrier family 48 member 1, SLC46A1 solute carrier family 46 member 1, SLC7A11 solute carrier family 7 member 11, SLC3A2 solute carrier family 3 member 2, SLC11A2 solute carrier family 11 member 2.

Two pivotal molecules in the amino acid metabolism of ferroptosis are GPX4 and system xc- (Fig. 1). GPX4 belongs to the GPX family and contains selenocysteine in its catalytic center, which is the only enzyme in the cell that can reduce lipid hydroperoxides to corresponding alcohols. When hydroperoxide isn’t effectively removed by GPX4, it accumulates further in the presence of iron, contributing to cell death [44]. As an essential intracellular antioxidant, reduced GSH is synthesized by glutamate, cysteine, glycine, and GSH synthase via the ATP-dependent cytoplasmic enzymes glutamate-cysteine ligase (GCL) and glutathione synthase (GSS). The availability of cysteine limits GSH synthesis. It has been demonstrated in vivo and in vitro that both extracellular cystine, which could be ingested and then transformed into cysteine, and tracellular cysteine is necessary for constraining GSH biosynthesis and for suppressing certain modes of death in mammalian cells that can be inhibited by iron-chelating agents [45–47]. For the synthesis of GSH, in addition to the above pathways, cysteine has been demonstrated to be absorbed from the environment through neutral amino acids to form an oxidized form through the system xc- cysteine/glutamate antiporter, or to be synthesized in the transsulfuration pathway (Fig. 1) [48].

#### Lipid peroxidation

In the 1950s, vitamin E and cysteine, as well as the trace element selenium, were certified as the bond that restrained lipid peroxidation [49–52]. Lipid peroxidation is evoked by a complicated lipid metabolism engaging initiation, propagation, and termination. Polyunsaturated fatty acids, such as arachidonic acid, are prime targets for peroxidation. Ferroptosis potentially proceeds through the peroxidation of membrane phospholipids to generate PLOOH and the breakdown of PLOOH to produce 4-hydroxynonenal or malondialdehyde. Products of this cascade reaction involving the decomposition products of lipid peroxides as well as oxidized or modified proteins, could cause membrane instability and permeabilization, ultimately bringing about cell death [53, 54]. In non-enzymatic lipid peroxidation, polyunsaturated fatty acids (PUFAs) are attached to coenzyme A (CoA) via the function of acyl-CoA synthase long-chain family member 4 (ACSL4) to generate acyl-CoA (Fig. 1). Subsequently, acyl-CoA could be re-esterified in phospholipids via lysophosphatidylcholine acyltransferase 3 (LPCAT3) to form PL. Regarded as crucial mediators of PUFA-PL composition and vital drivers of ferroptosis, the two membrane-remodeling enzymes, ACSL4 and LPCAT3, were uncovered from the Genome-wide haploid and CRISPR–Cas9-based screens. Regulation of ACSL4 and LPCATs may determine susceptibility to ferroptosis [54–61].

#### Iron metabolism

From the term “ferroptosis”, iron metabolism serves a vital function in the death of ferroptotic cells, including iron uptake, utilization, storage, and efflux [62]. Cells generally maintain iron homeostasis through orchestrated regulation and then impact the sensitivity of ferroptosis. The binding of iron-containing transferrin (TF) to transferrin receptor (TFR) and transferrin endocytosis could mediate the cellular iron uptake (Fig. 1) [63]. As for the storage of iron, numerous cellular processes could modulate the susceptibility to ferroptosis via altering the labile iron pool (LIP) (Fig. 1). In 2016, it was found that autophagy could facilitate ferroptosis by decomposing iron-storage protein ferritin in fibroblasts and cancer cells [64, 65]. Consequently, regulation of autophagy, including nuclear receptor coactivator 4 (NCOA4) and glutamate oxaloacetate transaminase 1 (GOT1) could increase or decrease LIP (Fig. 1) [65, 66]. The increasing cellular LIP could produce PLOOH and ROS directly or indirectly resulting in ferroptosis [67, 68]. Alternatively, augmenting cellular iron export is proven to enhance cellular resistance to ferroptosis [69, 70].

#### Mitochondrial mechanism

For lipid peroxidation and ferroptosis, the generation of mitochondrial reactive oxygen species (ROS) is crucial. As an essential supplier of ROS, mitochondria could generate hydroxyl radicals via the Fenton reaction, and then drives the PUFA-FL peroxidation [71]. The mitochondrion is also the major organelle to produce ATP through electron transport and proton pumping [72, 73]. Mechanically, with ATP-depletion, AMP-activated protein kinase (AMPK) could deactivate and phosphorylate Acetyl-CoA carboxylase (ACC), thus inhibiting PUFA-FL composition and ferroptosis, while under adequate cellular energy, PUFA-FL synthesis and ferroptosis are triggered. Therefore, ATP production contributes to ferroptosis [74, 75]. Mitochondria also have a hand in promoting ferroptosis with its function in biosynthetic pathways. Taken together, the versatile capabilities of mitochondria in bioenergetics and biosynthesis could trigger lipid peroxidation to ferroptosis. Controversially, Gao et al. have proved that mitochondria play a critical role in cysteine-depletion-induced ferroptosis but not in GPX4 inhibition-induced ferroptosis [76].

### The regulation pathway toward ferroptosis

#### Hippo-YAP signaling

The hippo-YAP pathway in ferroptosis, which could be involved in multiple biological functions such as cell proliferation, was identified by the heightened resistance of densely grown cells to cysteine deficiency- and GPX4 degradation-induced ferroptosis [59, 77]. Mechanistically, YAP targets several modulators of ferroptosis, such as ACSL4 and transferrin receptor 1 (TFR1). Hence, it is inevitable that ferroptosis susceptibility depends on the Hippo-YAP pathway, with the activation of Hippo signaling and the transcriptional co-regulator YAP-depletion [59].

#### AMPK

Energetic loss induced by the energy and metabolic disorder could bring about a serial breakdown of systems required to retain homeostasis, consequently contributing to cell death [78, 79]. Strikingly, glucose starvation plays an opposite role in influencing ferroptosis, either improving ferroptosis by an accumulation of ROS or blocking ferroptosis [74, 75]. The protective function was proved to rely on AMPK, a kinase sensing energy. Hence, stimulated by the absence of glucose, AMPK then turns on an energy stress-protective program against ferroptosis, impairing the biosynthesis of PUFAs [74, 80, 81].

#### HIF

Hypoxia-inducible factor (HIF) is composed of an oxygen-depleted α-subunit consisting of HIF1α, EPAS1 (HIF2α), and HIF3α, and a constitutively expressed β-subunit (ARNT) [82]. In specific circumstances, HIF1α and EPAS1 would perform differently. The expression of HIF1α and EPAS1 is upgraded in numerous cancer cell lines and is associated with inferior prognosis [82].

HIF possesses a double capacity in modulating ferroptosis [83]. Hypoxia-induced HIF1α impairs ferroptosis in fibrosarcoma by elevating fatty acid-binding proteins 3 and 7 levels to avert unrestrained lipid peroxidation [84]. Conversely, EPAS1-activation is involved in hypoxia-induced lipid droplet-associated protein (HILPDA) (Fig. 1) expression in RCC-derived cells, leading to ferroptosis [60]. Accordingly, effective regulation of HIF to sustain lipid homeostasis and thereby generate ferroptotic responses is essential.

#### EMT

The epithelial-mesenchymal transition (EMT) is a cellular process whereby epithelial cells repress characteristics, such as the polarity and intercellular adhesion, and progressively attain migratory and invasive faculties relevant to the mesenchymal phenotype [85].

In clinical practice, EMT is considered a cellular process to yield stem cells causing tumor metastasis and treatment resistance, for instance, SNAI1, TWIST1, and ZEB1 [85]. Equivalently, EMT is also concerned with inducing ferroptosis. Highly mesenchymal-like cell states are generally more sensitive to ferroptosis than those with epithelial properties [11, 13, 59]. ZEB1 is believed to function essentially in ferroptosis induction, partly attributed to the direct transcriptional regulation of PPARγ which primarily regulates lipid metabolism in the liver [86]. Regarded as a positive modulator of EMT, protein LYRIC (also known as metadherin) sustains ferroptosis by depleting GPX4 and SLC3A2 [87]. In summary, EMT might result in susceptibility to therapies based on ferroptosis.

### Anti-ferroptosis system

Ferroptosis is a critical anti-tumor mechanism. Tremendous evidence suggests that the tumor has evolved at least three mechanisms to avoid ferroptosis, such as downregulating PUFA-PL levels and lipid peroxidation, astricting the storage of iron in LIP, and uprising defense systems against ferroptosis.

#### The GPX4-GSH system

The antioxidant enzyme GPX4 is the only GPX member that straightway converts phospholipid hydroperoxide to phospholipid hydroxyphospholipid (Fig. 1), serving as a prime suppressor under diverse in vitro and in vivo conditions [14, 15, 22, 59, 88]. The activation of GPX4 depends on its cofactor, GSH, which provides electrons to GPX4 [89, 90]. Sometimes the electrons can be rendered by other low-molecular or protein thiols [91]. GSH is a thiol-containing tripeptide produced from cysteine, glycine, and glutamate, with cysteine being the major rate-astricting precursor. The majority of tumor cells acquire cysteine primarily utilizing system xc- with a transporter submit, the solute carrier family 7 member 11 (SLC7A11, also termed xCT) [92–94]. Depleting cysteine from the culture medium or blocking SLC7A11 through ferroptosis inducers (FINs) (Table 2), such as erastin, could trigger potent ferroptosis [5, 11, 95–97]. Therefore, SLC7A11-GSH-GPX4 is identified as the principal ferroptosis defense system.

**Table 2:** Ferroptosis inducers (FINs).

Class	Class characteristics	Compounds example	Suitable for in vivo use
i	GSH depleting compounds [178, 238]	Erastin, PE, IKE, other erastin analogs, sulfasalazine, sorafenib, glutamate [242, 243]	PE, IKE, sorafenib
ii	GPX4 inhibitors [13]	RSL3, ML162, DPI compounds 7,10,12,13,17,18,1	No [244]
iii	Depletion of GPX4 protein and CoQ10 [239]	FIN56, CIN56	Not evaluated yet
iv	Iron oxidation and indirect GPX4 inactivation [240, 241]	FINO2	Not evaluated yet

*CIL56* caspase-independent lethal 56, *CoQ10* coenzyme Q10, *DPI* diverse pharmacological inhibitor, *FIN56* ferroptosis inducer 56, *FINO2* ferroptosis inducer endoperoxide, *IKE* imidazole ketone erastin, *ML162* Molecular Libraries 162, *PE* piperazine erastin, *RSL3* RAS-selective lethal 3.

#### The FSP1–CoQH2 system

Ferroptosis suppressor protein 1 (FSP1), with its plasma membrane localization being a prerequisite for its function, operates as a NAD(P)H-dependent oxidoreductase capable of converting CoQ to CoQH2 [14, 15, 98]. CoQH2 could recruit lipid peroxyl radicals thus inhibiting lipid peroxidation and ferroptosis (Fig. 1).

#### The DHODH–CoQH2 system

A recent study unveiled a dihydroorotate dehydrogenase (DHODH)-mediated ferroptosis defense system in mitochondria that could compensate for GPX4 deficiencies to antitoxify mitochondrial lipid peroxidation [18]. Rendering with the substrate and product of DHODH could attenuate or potentiate ferroptosis induced by inhibition of GPX4 [18]. As GPX4 is sharply depleting, flux through DHODH noticeably rises, contributing to augmented production of CoQH2 which neutralizes extensive ferroptosis (Fig. 1) [18].

#### The GCH1–BH4 system

GTP cyclohydrolase 1 (GCH1) is also identified as a conditioner of ferroptosis [16, 17]. BH4 is capable of trapping lipid peroxyl radicals with GCH1 mediating the rate-limiting reaction in its biological synthesis [99]. Specifically, the GCH1-BH4 system suppresses ferroptosis through GCH1-mediated generation of CoQH2 as well as via the radical-trapping antioxidant ability of BH4 (Fig. 1) [16, 17].

#### The FSP1-Vitamin K system

The finding of the antioxidant effect of vitamin K predates the term ferroptosis, which has recently been demonstrated to be essential in anti-ferroptotic activity [100, 101]. All three forms of vitamin K, phylloquinone, menaquinone-4 (MK4) and menadione, could dampen ferroptosis through their reduced forms VKH2 [102]. The FSP1 could sustain a warfarin-insensitive non-canonical vitamin K cycle, suppressing ferroptosis by preserving VKH2 at the sacrifice of NAD(P)H to avoid lipid peroxidation [103].

### Ferroptosis vulnerability

#### Metabolic status and gene mutation in cancer cells

In certain cancer cells, not only the specific cellular states but also mutations in some genes will exhibit unexpected sensitivity to ferroptosis. As for the metabolic features, therapy-refractory cancer cells in certain statuses could perform surprisingly sensitive to ferroptosis [58, 104, 105]. Cancer cells with mesenchymal phenotype are highly dependent on GPX4 and are abundant in polyunsaturated fatty acids due to the elevated levels of ZEB1, ELOVL5, and FADS1 expression [58, 106, 107]. The sensitivity of dedifferentiated melanoma cell subtypes to ferroptosis owing to the accumulation of polyunsaturated fatty acids and the deficiency of GSH [58]. Similarly, certain cancer types with unique metabolic characteristics are susceptible to ferroptosis, such as triple-negative breast cancer (TNBC), clear-cell RCC (ccRCC), and non-neuroendocrine small cell lung cancer (SCLC). TNBC cells could elevate the levels of PUFA and LIP while attenuating the GPX4-GSH defense system [55, 108].

Additionally, genetic mutations, for instance, the inactivation of tumor suppressor genes in certain cases, also increase susceptibility to ferroptosis. Incapacitation of any element of the E-cadherin-NF2-Hippo pathway contributes to the expression or activity of its downstream molecule YAP, leaving cancer cells or mutant tumors like NF2- mutant mesotheliomas especially vulnerable to FINs [59, 109].

#### The imbalance in the ferroptosis defense system

Ferroptosis can be suppressed by GPX4-dependent and GPX-independent methods. Once one method is inactivated or exhausted, cancer cells become extremely reliant on the other method to fight against ferroptosis and are supremely sensitive to ferroptosis induced by the other defense system.

### Immune tolerance mediated by ferroptosis

Inducing and maintaining robust immune tolerance have been the holy grail of immunology for decades. How to effectively restrain tumor immune tolerance still requires long-term investigation.

Peripheral immune tolerance can be induced by the co-stimulatory or co-inhibitory signaling pathway, equally vital as which, additional controls on the surface of activated T cells, acknowledged as immune checkpoints, can also generate active immune tolerance. Another key mechanism is the presence of specialized cell populations designed to suppress pathogenic immune responses that inadvertently target self-tissue [110]. Ferroptosis obtains a double-sided effect on regulating tumor immune tolerance. Hence, we focus on the role of ferroptosis in mediating tumor peripheral immune tolerance via the three pathways mentioned below.

#### Co-stimulatory or co-inhibitory receptor

**Fig. 2:**
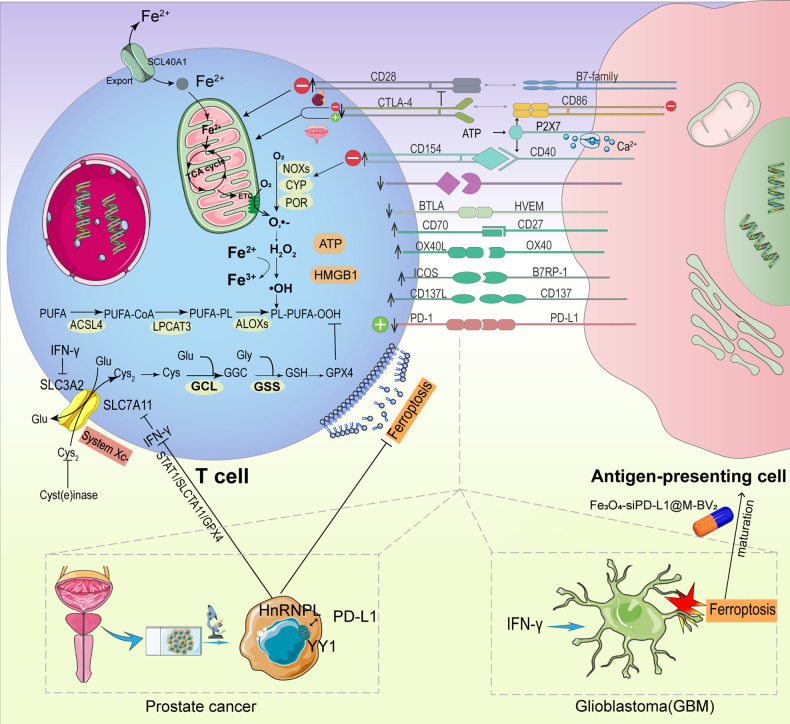
Co-stimulatory, co-inhibitory and checkpoint pathways. In addition to the co-stimulatory, co-inhibitory and checkpoint pathways, there are other stimulatory and inhibitory pathways (respectively indicated by upward and downward arrows,) that impact the immune response, including tumor necrosis factor (TNF)-related molecules, other members of the CD28 family, adhesion molecules, and T-cell immunoglobulin and Mucin (TIM) molecules. Various stimulatory and inhibitory pathways can affect the onset of ferroptosis in immune cells and tumor cells via a wide range of mechanisms, ultimately facilitating (green plus) or inhibit (red minus) immune tolerance. Moreover, prostate cancer cells could upregulate PD-L1 through HnRNPL over-expression, which in turn inhibits IFN-γ released by CD8 + T cells via the STAT1/SLC7A11/GPX4 signaling axis. Subsequently, the expression of SLC3A2 and SLC7A11 (two subunits of system Xc-) increases, suppressing lipid peroxidation by facilitating cystine uptake, which ultimately contributes to ferroptosis evasion and dampens tumor immunity. Likewise in GBM, activated CD8 + T cells could release IFN-γ, inducing ferroptosis in cancer cells. Fe3O4-siPD-L1@M-BV2, a novel GBM-targeted pharmaceutical delivery system, could stimulate ferroptosis for immunotherapy of drug-resistant GBM and establish a cascade of amplification between ferroptosis and immune activation.

Immune cell functions are regulated by co-inhibitory and co-stimulatory receptors. Effective activation of these receptors may have significant therapeutic benefits and prospects in anti-cancer immunity [111–114]. Ferroptosis, as a non-apoptotic iron-dependent form of cell death, is predicted to be profitable in inhibiting cancer immune tolerance mediated by apoptotic immune cell death. Thus, in the tumor immune microenvironment, the combination of co-stimulatory or co-inhibitory signals and ferroptosis could achieve a greater clinical therapeutic prospect promisingly (Table 3). The key to success lies in precise comprehension of the correlation, aiming to ameliorate immune tolerance maximally (Fig. 2).

**Table 3:** Potential clinical implications of ferroptosis mediating immune tolerance.

Pathway	Target	Significance	Relationship between ferroptosis and immune tolerance	Mechanism	Potential therapies	Target of agent	References
Co-stimulatory recepto	CD28	GPX4 deficient Tregs contribute to ferroptosis upon T cell receptor/CD28 co-stimulation.	Ferroptosis may obstruct immune tolerance.	Induce the death of GPX4 deficient Tregs through the CD28 costimulation pathway.	Take the cancer cells with imbalanced ferroptosis defense system, GPX4 low, as target.	FSP1 inhibitors, DHODH inhibitors, GCH1 inhibitors, BH4 depletion.	[14, 16–18, 32, 38, 115, 116]
	CTLA-4	The expression of CTLA-4 is higher in high ferroptosis scores group, but its meaning varies.	The effect of immune tolerance mediated by upregulation of CTLA-4 via ferroptosis varies in diverse tumors, which still needs to be specifically analyzed in extensive investigations	The blockade of CTLA-4	Combined induction of ferroptosis and immune checkpoint inhibitors.	-	[40, 42, 116, 118]
	CD40	CD40- CD154 is the core of humoral immune response	Ferroptosis could inhibit immune tolerance	CD40-ATP-PX27 pathway	Inducing ferroptosis	FINs	[125, 127–134]
	CD86	The mobilization of ATP-P2X7-CD86 axis ultimately intensified T cell activation.	Ferroptosis could inhibit immune tolerance	ATP-P2X7-CD86 pathway	Inducing ferroptosis	FINs	[142, 144–148]
Immune checkpoint	PD-1, PD-L1	The expression of PD-L1 has different meanings. Poor prognosis in multiple cancers including RCC and ovarian canecer. Optimistic prognosis in breast cancer and Merkel cell carcinoma.Dual value in CRC and melanoma	Ferroptosis could either inhibit or promote immune tolerance	Prostate cancer. PD-L1 is related to HnPNPL. HnRNPL knockdown could inhibit the expression of PD-L1, thus producing increased IFN-γ, which triggers ferroptosis of CRPC cells by the STAT1/SLC7A11/GPX4 signaling axis	Overcoming resistance to conventional cancer therapies	TYRO3 inhibitors+ anti- PD1	[155–157, 159–163, 165–168, 176, 178, 181–184, 188–190]
						Fe3O4-siPD-L1@M-BV2	
						GW4869-meditated PD-L1-based exosomes	
						zero-valent-iron nanoparticles (ZVI-NP)	
Specialized cell population	TGF-β-producing Th3 cells	TGF-β 1 could enhance ultrastructural variation in mitochondria with increased ROS and MDA	Ferroptosis may obstruct immune tolerance	GSH level and the lipid peroxidation	Prohibiting TGF-β induced ferroptosis	Low GSH level and enhanced lipid peroxidaton	[193–201]
		TGF-β 2 could affect the expression of GPX4 and ACSL4

#### CD28

Effective T cell signaling requires both participation of primary antigen-specific receptors and a second costimulatory signal to generate proliferation, differentiation, and survival [115]. As a constitutive T-cell-surface molecule, CD28 is the first T-cell costimulatory receptor to be identified. B7-CD28 costimulation could facilitate thymus central immune tolerance by forming T cell libraries and limiting autoimmune functions through regulatory T cell (Treg) generation. Without B7-CD28 costimulation, Treg generation is defective, while functional self-reactive T cells accumulate in the periphery, mediating destructive autoimmunity, which restrains immune tolerance consequently [32, 116]. Further, Gpx4-deficient Tregs can contribute to ferroptosis upon T cell receptor (TCR)/CD28 co-stimulation, thus weakening immune tolerance [38]. Hence, ferroptosis may obstruct immune tolerance by inducing the death of Gpx4-deficient Tregs through the CD28 costimulation pathway.

#### CTLA-4

CTLA-4 blockade contributed to the CD28-mediated proliferation of tumor-associated Tregs, inducing tumor immune tolerance ultimately. This procedure allows polyclonal Treg repertoire to be pre-enriched for recognizing antigens submitted by tumor-associated conventional dendritic cells (CDCs). Unsteady CDC contacts ensured Treg capability, while CTLA-4-mediated downregulation of CDC co-stimulatory B7 proteins, via Tregs, resulted in contact instability. CTLA-4-blockade sparked CD28-dependent Tregs hyper-proliferation in the TME. Therefore, the blockade of CTLA-4 may lessen therapeutic benefits in cancer patients [116].

Studies revealed that CTLA-4 expression was higher in high ferroptosis scores groups than in the lower ones. In bladder cancer, low ferropscores indicated a better immunotherapy response, while in clear cell renal cell carcinoma, the high ferropscores were characterized by a higher immune and enhanced response to anti-CTLA-4 immunotherapy [117, 118]. Accordingly, the effect of immune tolerance mediated by upregulation of CTLA-4 via ferroptosis varies in diverse tumors, which still needs to be specifically analyzed in extensive investigations. Further, combined induction of ferroptosis and immune checkpoint inhibitors (ICIs) indicated synergistic anti-tumor activity, providing significant avenues for future research might and potentially driving immunotherapy of cancer to promising results, especially on PD-1 and CTLA-4 targets [40, 42].

#### CD40

CD40 is involved in humoral immunity and inflammatory response. Belonging to the tumor necrosis factor (TNF) receptor superfamily, CD40 is expressed in a variety of cells, including antigen-presenting cells (APCs) and fibroblasts [119–124]. CD154, the receptor of CD40, mainly locates on activated CD4 + T cells [125, 126]. The CD40-CD154 pathway is the core of humoral immune response, simultaneously promoting dendritic cell (DC) maturation and inducing effective T cell initiation [125, 127, 128]. The CD40-ATP-P2X7 pathway can amplify inflammation and contribute to the death of retinal endothelial cells. It was established that CD40 was a novel generator of ATP release and purine-induced inflammation. By engaging the P2X7 receptor, ATP subsequently induces the release of pro-inflammatory cytokines by monocytes, macrophages, and microglia, resulting in the death of retinal endothelial cells [129, 130]. Additionally, CD40 participates in the pathological process of autoimmune, and anti-CD40 monoclonal antibodies can lead to immune tolerance [131–134].

As the most well-characterized damage-associated molecular patterns (DAMPs) implicated in immunogenic cell death, ATP and high mobility group box chromosomal protein 1 (HMGB1) have been elucidated to be automatically emitted following the timeline of ferroptosis, which also serve as immune signals correlated with the immunogenicity of early ferroptotic cancer cells. Further, ATP and HMGB1 may be essential in accelerating ferroptotic immunogenic cell death (ICD) [135]. Therefore, ATP participates as an immunogenic signal of ferroptosis, while ferroptosis could inhibit immune tolerance through the CD40-ATP-P2X7 pathway promisingly. Given the above, ferroptosis is proposed as a potential immunogenic mode of cell death [136–140]. Nevertheless, Wiernicki et al. held a contradictory perspective, asserting that ferroptotic DCs were unable to resist tumor growth compared to necroptosis and apoptosis, and that ferroptotic cancer cells entirely failed to trigger immune protection in spite of releasing DAMP and cytokines. Ferroptosis adversely affected antigen-presenting cells and the adaptive immune response, which might also interfere with cancer immune treatments, hampering ferroptosis-induced therapeutic applications [141]. Thus, whether ferroptosis is indeed immunogenic still demands additional work to demonstrate experimentally for a better understanding of its role in anti-cancer therapy.

#### CD86

CD86 could prohibit immune tolerance [142]. As research on hypertension revealed, the mobilization of the ATP-P2X7-CD86 axis ultimately intensified T cell activation. Hypertension generates an accumulating release of ATP in plasma. ATP initiates revitalization of APCs, which in turn stimulates P2X7 receptors on APCs, increasing expression of CD86 through P2X7-gated Ca2+ influx and activating T cells ultimately [143]. There are compact links between cancer and hypertension on account of their overlapping risk factors and pathophysiological mechanisms [144–146]. Extracellular ATP has been certified to modulate the expression of P2X7 receptors on APCs in cancer [147, 148]. Thus, the ATP-P2X7-CD86 axis might function in tumors up-regulating CD86. Ferroptosis could ultimately inhibit immune tolerance through the ATP-P2X7-CD86 axis.

### Immune checkpoint

Immunotherapy is a promising strategy to treat malignant tumors by harnessing the cytotoxic potential of the immune system, especially tumor-specific cytotoxic T cells. ICIs have an extensive impact and early success in the clinic. Such as PD-1 ligand (PD-L1) within TME, the expression of immune checkpoint molecules has been shown to predict response to ICIs in some but not all cases [149, 150]. Some patients with PD-L1-positive tumors don’t react to treatment, while some patients with PD-L1-negative tumors may also benefit from ICIs [41, 151, 152]. Ferroptosis is intensely associated with anti-tumor immunity and immune microenvironment [153, 154], and is of great significance in improving traditional drug resistance. Hence, the combination of ferroptosis and ICIs could be a new exploration for reducing ICIs resistance and ultimately broaden the pool of patients potentially benefiting from ICIs (Table 3).

#### PD-1 and PD-L1

PD-1 and PD-L1 are significant proteins for immune regulation and have a dual impact on cancer prognosis [155]. PD-L1 expression strongly predicted poor prognosis in multiple cancers, including RCC and ovarian cancer [156–161], while optimistic prognosis has been confirmed in breast cancer and Merkel cell carcinoma [162, 163]. Surprisingly, in CRC and melanoma, the simultaneously positive and negative predictive value of PD-L1 expression was observed [164–169]. Hence, there is an urgent need to seek strategies to improve the traditional PD-1 and PD-L1 targeting immunotherapies. Coded by the CD274 gene, PD-L1 is the transmembrane protein that can conduce to immunosuppression by combining with PD-1 presented on T cells and eliciting T-cell asthenia [170]. The pool of PD-L1 has been implicated in being enlarged to evade immune surveillance in lung cancer and bladder cancer [171–175].

In prostate cancer, PD-L1 was positively related to heterogeneous nuclear ribonucleoprotein L (HnRNPL) [176], which overexpressed and consequently enhanced the mRNA stability of YY1, in turn generating pro-proliferative and anti-apoptotic effects [177]. HnRNPL knockout effectively downregulates PD-L1 and restores the cancer cell sensitivity to T cell killing in vitro and in vivo. Ferroptosis functions remarkably in T cell-driven adverse outcomes of cancer cells, and HnRNPL inhibits Jurkat T cell-mediated Castration-Resistant Prostate Cancer (CRPC) cell ferroptosis through the YY1/PD-L1 axis, partially promoting cancer immune escape. Further, suppression of HnRNPL boosted the anti-PD-1 effect of CRPC tumors via enrolling CD8 + T cells in PCa tumors [176]. Investigations have revealed that ferroptosis is a previously unrecognized mechanism of CD8 + T cell-mediated tumor elimination [178]. IFN-γ is one of the primary cytokines released by effector CD8 + T cells [179, 180]. IFN-γ down-modulates the expression of SLC3A2 and SLC7A11 and inactivates cystine uptake, in turn promoting lipid peroxidation and ferroptosis in tumor cells and increasing the clinical benefit of cancer immunotherapy [178]. HnRNPL knockdown could inhibit the expression of PD-L1, thus producing increased IFN-γ, which triggers ferroptosis of CRPC cells by the STAT1/SLC7A11/GPX4 signaling axis [176, 178].

Glioblastoma (GBM) is an invasive intracranial malignant tumor [181]. Frustratingly, with long-term temozolomide (TMZ)-therapy in GBM patients, drug resistance ineluctably develops and the efficacy is remarkably attenuated or even eliminated [182, 183]. As the most abundant programmed cell death process in glioma [184], ferroptosis induces DCs maturation and enhances T cell activity. Activated T cells then release IFN-γ, inducing the ferroptosis of cancer cells. Thereby, a novel GBM-targeting pharmaceutical delivering system, Fe_3_O_4_-siPD-L1@M-BV2, was constructed to facilitate ferroptosis for immunotherapy of drug-resistant GBM. A cascade of amplification between ferroptosis and immune activation was formed via the system, ultimately prohibiting tumor growth [181].

Tumor-derived exosomes could restrain DC maturation, down-regulate the expression of surface markers like CD80, CD86, and MHC-II, and up-regulate the expression of CD11b and PD-L1, which ultimately obstructed the anti-tumor activity of Teff and reinforced immune evasion [185–187]. Xie et al. designed phototheranostic metal-phenolic networks (PFGMPNs) via a semiconductor polymer assembly that encapsulated FIN (Fe3+) and GW4869 (exosome inhibitor). More strikingly, GW4869-meditated PD-L1-based exosomes hinder revitalized T cells and amplify ferroptosis. This neoteric synergistic effect of photothermal therapy (PTT) with anti-exosomal PD-L1 enhanced ferroptosis and induced effective anti-tumor immunity in tumors [188].

Although immunotherapy with ICIs has generated significant positive applications in a subset of cancer patients, there are still numerous mechanisms of tumors to motivate drug resistance. It was revealed that TYRO3 prohibited the ferroptosis of tumor cells induced by anti-PD-1/PD-L1 via the AKT/NRF2 axis and amplified a pro-tumor microenvironment by downgrading the ratio of M1/M2 macrophages, consequently contributing to anti-PD-1/PD-L1 treatment [189]. Thus, TYRO3 could serve as a predictive biomarker and a promising therapeutic target for overcoming anti-PD-1/PD-L1 resistance in cancer patients. By understanding the specific mechanisms of drug resistance targeting immune checkpoints, it is of great promise to hinder drug resistance and enhance the efficacy of therapies that promote ferroptosis in tumor cells in combination with ICIs.

Simultaneous induction of cancer-target cytotoxicity and anti-tumor immunity could be a prospective method for treating drug-resistant tumors. An investigation highlighted the potential of zero-valent-iron nanoparticles (ZVI-NP) as an innovative integrative cancer-fighting strategy. Notably, ZVI-NP enhanced anti-tumor immunity via transforming pro-tumor M2 macrophages into anti-tumor M1, reducing Tregs and down-regulating CTLA-4 and PD-1 in CD8 + T cells to provoke their cytolytic activity against cancer cells. Therefore, the dual mechanism of anti-cancer activity of ZVI-NPs insightfully exploited the prospect of novel anti-cancer therapies while reducing adverse effects and improving prognosis [190]. To conclude, multiple studies have systematically demonstrated that synergy between ferroptosis and immunoregulation could generate a significant anti-cancer effect, showing great promise in anti-cancer therapy.

Nevertheless, ferroptosis in glioma might generate an immunosuppressive microenvironment, eventually contributing to immunotherapy resistance. Enhanced ferroptosis revealed the induction of immune cell activation and infiltration, but attenuated anti-tumor cytotoxic killing, among which tumor-associated macrophages (TAMs) were involved in ferroptosis-mediated immunosuppression. TAM was the most abundant immune cell in GBM tissue and initiated pro-inflammatory (M1) or immunosuppressant (M2) responses according to its polarization state. Patients with enhanced ferroptosis were characterized by recruitment of TAM and M2 polarization. The combination of ferroptosis obstacle with PD-1/L1 blockade triggered synergistic therapeutic results in GBM mouse models [184]. Hence, considering the dual role of ferroptosis in tumor cell death and immunosuppressive phenotypes, how to optimize the therapeutic benefits of ferroptosis in different cancers by balancing ferroptosis and overcoming immunosuppressive phenotypes still requires to be clarified.

### Specialized cell populations

Specialized cell populations include naturally occurring Treg and in vitro- induced Tregs (iTregs), as well as type 1 regulatory T (Tr1) cells and TGF-β producing type 3 helper T (Th3) cells. Of these subpopulations, the most extensively studied are Tregs [191]. By acquiring a better understanding of ferroptosis and specialized cell populations, it is feasible to combine them to promote immune tolerance antagonism (Table 3).

#### TGF-β-producing Th3

Th3 regulatory cells are a unique T cell subset that secretes TGF-β and assists IgA [192]. TGF-β functions in a variety of diseases by mediating ferroptosis, such as coronary heart disease, diabetes, acute liver failure (ALF), and pulmonary fibrosis. In mammals, there are three subtypes of TGF-β, including TGF-β1, TGF-β2, and TGF-β3 [193]. TGF-β1 enhances ultrastructural variation in mitochondria with increased ROS and MDA levels, similar to ferroptosis [194, 195]. Further, current research speculated that TGF-β2 affected the expression of GPX4, nuclear factor E2-related factor 2 (NRF2), HO-1, NOX4, and ACSL4 to promote ferroptosis [196–201]. Thus, the combination of TGF-β and ferroptosis could have a prospective therapeutic outlook and provide potential targets for cancer immunotherapy. XCT is associated with unfavorable prognosis for various types of tumors, including HCC, colorectal cancer, and GBM [202, 203]. In diabetes, TGF-β1 can induce renal tubular cell death, which contributes to diabetic nephropathy, as well as renal fibrosis. In TGF-β1-stimulated renal tubular cells, the expression of xCT and GPX4 as the same as the GSH level is dramatically decreased. The lipid peroxidation is enhanced adversely. Both the GSH and lipid peroxidation are associated with ferroptosis [204]. Therefore, prohibiting TGF-β1-induced ferroptosis in renal tubular cells may be a promising method for preventing or treating diabetic nephropathy. These findings are in line with the research showing that TGF-β1 enhances lipid peroxidation and inhibits susceptibility to GPX4 in hepatocellular cancer cells by repressing xCT expression via Smad3 activation. Moreover, TGF-β obtains a profound effect in the process of EMT in pulmonary fibrosis. Upregulating TGF-β amplifies ferroptosis and ultimately generates EMT [205, 206]. Hence, TGF-β could be applied as a promising target to amplify ferroptosis and reinforce EMT, ulteriorly achieving the purpose of cancer treatment. The combination of Fe, checkpoint antibody (Pa), and TGF-β inhibitor (Ti) with constructed nanoparticles (NPs) can effectively enhance immunogenic TME and kill tumor cells [207]. All these investigations could propose an underlying new method for future cancer treatment.

### Ferroptosis vulnerability of immune cells

Apart from cancer cells, TME also comprises immune cells, including T cells, macrophages, myeloid-derived suppressor cells (MDSCs), etc. [208], which are proven to obtain similar growth signals and metabolic properties to cancer cells [209–211]. This peculiarity contributes to the analogical vulnerability of immune and tumor cells to anti-tumor therapy, which could in turn impair the function of immune cells. T cells are crucial in anti-tumor immunity [211]. Ferroptosis can serve as the metabolic vulnerability of tumor-specific CD8 + T cells, while GPX4-deficient T cells render high sensitivity to ferroptosis, consequently being unable to exert an ant i-tumor effect. GPX4 overexpression inhibited ferroptosis in CD8 + T cells and restored the production of cytotoxic cytokines in vitro [212, 213], simultaneously increasing the quantity of cancer infiltrative CD8 + T cells in vivo, which enhanced tumor control [213, 214].

As a portion of CD4 + T cells, Tregs can hamper anti-tumor immunity. Tregs rapidly induce GPX4 expression after TCR/CD28 co-stimulation activation to avert ferroptosis [38]. Ferroptosis inhibitors can prohibit ferroptosis in activated GPX4-deficient Tregs and maintain their immunosuppressive function in tumors. Ferroptosis inhibitors may be a promising strategy to enhance anti-tumor immunity. Nevertheless, the effect is dissimilar due to differences in the sensitivity of tumor cells and CD8 + T cells to proferroptotic stimulation. It is of great necessity to further explore the effect of ferroptosis on tumor immunotherapy under different conditions.

MDSCs in TME have potent immunosuppressive capacity exhibiting resistance to ferroptosis [215]. Ferroptosis promotion of MDSC will be a promising target for improving tumor immunosuppressive microenvironment. TAMs predominantly present M2 phenotype to suppress anti-tumor immunity [216]. The resistance of M1 to ferroptosis was more intense than M2 [217]. Hence, eradicating M2 TAM or reverting M2 to anti-tumor M1 phenotype is of vital prospect for anti-cancer immunotherapy [218]. Evidence showed that elimination of GPX4 in TAM could restrain the viability of M2 TAM without affecting M1. Thus, targeting these cells with FINs is a potentially promising therapeutic strategy to reverse immunosuppressive TME (Table 2) [217].

It was widely validated that natural killer (NK) cells were also affected by the ferroptosis, resulting in dysfunction [219], and NRF2 could save NK cell glucose metabolism and anti-tumor activity in vivo [196, 219]. DCs, as specialized APCs demanded for naïve T cell function and maintaining T cell-dependent immunity, were also vulnerable to ferroptosis [220]. In diverse B-cell subsets with contrary features in tumor immunity, B1 and MZ B cells exhibited greater amounts of CD36 and absorbed greater amounts of lipids than follicular B2 cells. Deprivation of GPX4 provoked lipid peroxidation and triggered ferroptosis in B1 and MZ B cells, but not in follicular B2 cells [221]. Tumor cells may release various signals to stimulate or suppress ferroptosis in different immune cells. In sum, the vulnerability of immune cells to ferroptosis plays a dual role in anti-tumor immunity. For one thing, induction of ferroptosis may attenuate the survival of anti-tumor immune cells and contribute to functional defects. For another, some immunosuppressive immune cells require GPX4 to forestall ferroptosis and maintain cell activation. Simultaneously inhibiting ferroptosis of anti-tumor immune cells and promoting ferroptosis of immunosuppressive immune cells could maximize the benefits of tumor immunotherapy. How to balance the ferroptosis vulnerabilities of cancer cells, anti-tumor immune cells, and immunosuppressive cells remains a crucial encumbrance. A comprehensive exploration of the mechanisms behind the varying susceptibilities of cancer cells and diverse immune cells to ferroptosis is essential.

## Conclusions and challenges

In summary, ferroptosis, a non-traditional pattern of cell death, operates as a double-edged sword in mediating tumor immune tolerance. The mechanisms of ferroptosis in diverse tumor cells require further exploration to maximize clinical therapeutic benefits. Meanwhile, the susceptibility of tumor cells to ferroptosis, to some extent, implies that regulating ferroptosis could be a novel treatment for drug-resistant tumors.

Nevertheless, to fully recognize the potential of ferroptosis-inducing strategies in cancer therapy, there are still vast thought-provoking uncertainties to be clarified in future investigations. Firstly, how to target tumor types or patients more accurately that are considered suitable for proferroptotic therapy? How to detect the iron level of tumor cells? It was confirmed that appropriateness could be assessed by iron level, gene, and mutation levels in tumor cells [222, 223]. Iron-rich tumors such as breast cancer [55] and HCC [199] are more likely to benefit. Thus, the detection of these three factors is of great significance for the selection of therapeutic targets. Secondly, are there additional ferroptosis defenses in other organelles? The recently summited compartmentalization model suggested that extra ferroptosis defense systems might exist in other organelles [18]. The discovery of neoteric defense systems could be a promising solution to ferroptosis resistance. Thirdly, current studies have indicated additional ferroptosis enforcement mechanisms downstream of plasma membrane lipid peroxidation, but the specific steps remain to be discovered. The discovery of downstream steps may help identify new targets for cancer therapy. Fourth, more research is needed to explore other regulative mechanisms associated with ferroptosis execution and their correlation to anti-tumor therapy. Fifth, how to balance the vulnerability of ferroptosis among tumor cells, anti-tumor immune cells, and immunosuppressive cells? Meanwhile, how can pharmacological suppression of GPX4 specifically target tumors without causing wide-ranging virulence in patients’ normal tissues? Prophetic biomarkers that can precisely forecast tumor responsiveness to ferroptosis induction remain to be discovered, particularly those that can be detected directly in patient body fluids and biopsy specimens aiming to screen targeted patients and assess clinical efficacy. Via erastin-treated HT −1080 cells by RNA-Seq and RT-qPCR, the upregulation of the ER stress response gene cation transport regulator homolog 1 (CHAC1) was validated to be a significant pharmacodynamic marker of system xc- inhibition and a transcriptional PD marker for exposure to erastin and other agents that deplete cells of cystine or cysteine [83, 224, 225]. Identified by PCR, western blot assay, gene transfection, and ACSL4 knockdown techniques, ACSL4 was found as a biomarker and contributor of ferroptosis [226]. Feng et al. elucidated that 3F3 ferroptotic membrane antibody (3F3-FMA) could detect ferroptotic cells by screening antibodies that utilized TfR1 protein as antigen. And anti-TfR1 antibodies could detect ferroptosis by immunofluorescence and flow cytometry. 3F3-FMA was validated as a ferroptosis-detecting antibody in cell culture and cancer models [227]. Although some biomarkers of ferroptosis have been investigated, it is far from enough in clinical practice. Accordingly, novel technologies such as liquid biopsy, high-dimensional cytology, single-cell omics, metabolomics, and high-resolution imaging could be used to discover more easily detected biomarkers, bringing great convenience to drug-resistant cancer treatment.

To sum up, ferroptosis will be an extremely promising research direction and can provide a new method for drug-resistant cancer treatment. It will generate massive clinical benefits only if balancing the double-sided effect of ferroptosis in the tumor immune microenvironment.

## Data Availability

All data generated in the current study are available.
